# Supplementary material for: The use of digital technology in non-pharmacological cognitive and psychosocial interventions for people with dementia and mild cognitive impairment: A scoping review
Source: PLoS One. 2026 Apr 30;21(4):e0346008. doi: 10.1371/journal.pone.0346008 (PMC13132441; doi:10.1371/journal.pone.0346008)
Supplement: S1 Table — (PDF) [file pone.0346008.s001.pdf]

Database	Search String
Scopus	*(“cognitive training” OR "cognitive intervention" OR “psychosocial intervention*” OR “cognitive stimulation”) AND (digi* OR computer* OR web* OR technolog*) AND (“dementia” OR “Alzheimer*” OR "MCI" OR "mild cognitive impairment")*
Pubmed	(("cognitive training"[Title/Abstract] OR "cognitive intervention"[Title/Abstract] OR "psychosocial intervention*"[Title/Abstract] OR "cognitive stimulation"[Title/Abstract]) AND (digi*[Title/Abstract] OR computer*[Title/Abstract] OR web*[Title/Abstract] OR technolog*[Title/Abstract])) AND ("dementia"[Title/Abstract] OR "Alzheimer*"[Title/Abstract] OR "MCI"[Title/Abstract] OR "mild cognitive impairment"[Title/Abstract])
Dimensions	(“cognitive training” OR "cognitive intervention" OR “psychosocial intervention*” OR “cognitive stimulation”) AND (digital OR digitized OR digitised OR digitalized OR digitalised OR computer OR computing OR computerised OR computerized OR web OR "web-" OR technology OR technologies OR technological) AND (“dementia” OR “Alzheimer*” OR "MCI" OR "mild cognitive impairment")
Cinahl	(AB ((“cognitive training” OR "cognitive intervention" OR “psychosocial interventions” OR “cognitive stimulation”)) OR TI ((“cognitive training” OR "cognitive intervention" OR “psychosocial interventions” OR “cognitive stimulation”))) AND (AB ((digi* OR computer* OR web* OR technolog*)) OR TI ((digi* OR computer* OR web* technolog*))) AND (AB ((“dementia” OR “Alzheimer*” OR "MCI" OR "mild cognitive impairment")) OR TI ((“dementia” OR “Alzheimer*” OR "MCI" OR "mild cognitive impairment")))
Embase	('cognitive training':ab,ti OR 'cognitive intervention':ab,ti OR 'psychosocial intervention*':ab,ti OR 'cognitive stimulation':ab,ti) AND (digi*:ab,ti OR computer*:ab,ti OR web*:ab,ti OR technolog*:ab,ti) AND ('dementia':ab,ti OR 'alzheimer*':ab,ti OR 'mci':ab,ti OR 'mild cognitive impairment':ab,ti) AND [2012-2022]/py AND [embase]/lim
PsycInfo	(“cognitive training” OR "cognitive intervention" OR “psychosocial intervention*” OR “cognitive stimulation”) AND (digi* OR computer* OR web* OR technolog*) AND (“dementia” OR “Alzheimer*” OR "MCI" OR "mild cognitive impairment")
