# Supplementary material for: The use of digital technology in non-pharmacological cognitive and psychosocial interventions for people with dementia and mild cognitive impairment: A scoping review
Source: PLoS One. 2026 Apr 30;21(4):e0346008. doi: 10.1371/journal.pone.0346008 (PMC13132441; doi:10.1371/journal.pone.0346008)
Supplement: S2 Table — (PDF) [file pone.0346008.s002.pdf]

S3: Appendix

	Country	Technology								
		APP	BCI	DESK	FOOT	GT	ROB	VR	WEB	Grand Total
**Asia**	China	2				1		4	1	8
	Hong Kong	1		1		1				3
	India	1				1				2
	Israel	1		1						2
	Japan	1								1
	Jordan	1								1
	Pakistan					1				1
	South Korea	8		4		7	1	10		30
	Taiwan			1		3		3	3	10
	Turkey	1							1	2
	**Asia Total**	**16**		**7**		**14**	**1**	**17**	**5**	**60**
**Europe**	Austria	1								1
	Estonia							1		1
	France	1		1		2	1		2	7
	Germany	3		1		3				7
	Greece	2				2		2	1	7
	Italy	3	2	3	1	1	1	3	2	16
	Netherlands					1		1	1	3
	Norway	1				1				2
	Poland					1		1	2	4
	Portugal	1				1		2	2	6
	Slovakia					1				1
	Spain	2		1		3			4	10
	Switzerland					1				1
	UK	3		1		2				6
	**Europe Total**	**17**	**2**	**7**	**1**	**19**	**2**	**10**	**14**	**72**
**North America**	Canada	1								1
	Mexico			1		1		1	1	4
	USA	5	1	4		3			3	16
	**N. A. Total**	**6**	**1**	**5**		**4**		**1**	**4**	**21**
**Oceania**	Australia			1		1			3	5
	New Zealand						1			1
	**Oceania Total**			**1**		**1**	**1**		**3**	**6**
	**Grand Total**	**39**	**3**	**20**	**1**	**38**	**4**	**28**	**26**	**159**

Table 5. Types of technology used per country and per region.
