# Supplementary material for: The use of digital technology in non-pharmacological cognitive and psychosocial interventions for people with dementia and mild cognitive impairment: A scoping review
Source: PLoS One. 2026 Apr 30;21(4):e0346008. doi: 10.1371/journal.pone.0346008 (PMC13132441; doi:10.1371/journal.pone.0346008)
Supplement: S3 Table — (PDF) [file pone.0346008.s003.pdf]

Study	Outcome Summary
Abdalrahim et al. (2022)	Not specified; Other
Alves et al. (2019)	Not specified; Other
Amjad et al. (2019)	Cognitive (Global cognition:↑↑ Attention/Executive Function: ↓↓)
An et al. (2025)	Cognitive (Global cognition: ↑ Attention: 一 ↑)
Bahar-Fuchs et al. (2017)	Cognitive (Global cognition: ↑ Memory: ↑ ↑) Functional (↑)
Baik et al. (2023)	Cognitive (Global cognition: ↑ Memory/Learning: ↑↑ Executive Function: ↑↑) Well-Being (↑)
Baldimtsi et al. (2023)	Cognitive (Global Cognition: ↑ Excutive Function: 一 Memory: ↑一)
Bamidis et al. (2015)	Cognitive (Global Cognition: ↑ Memory: ↑ Executive Function: ↑ Working Memory 一) Well-Being (一) Functional (一↑)
Barban et al. (2016)	Cognitive (Memory: ↑ Executive Function: 一)
Ben-Sadoun et al. (2016)	Cognitive (MMSE: 一; FAB; 一; SCB Fluency: 一; SCB Memory: 一; TMT A: 一; TMT B: ↑; DSST: 一; DMS48; 一; X-Torp TMT A: ↑; X-Torp-TMT B: ↑; X-Torp DSST: ↑; X-Torp DMS48: ↑; X-Torp CT: ↑一; X-Torp GNGRTT: 一一)
Bernini et al. (2023)	Not specified; other (high motivation of use, usability ratings)
Brem et al. (2020)	Cognitive (Global cognition: ↑)
Burgos-Morelos et al. (2025)	Cognitive (Sensory-motor integration: ↑↑)
Caroppo et al. (2017)	Not specified; other (postive attitude to games)
Cavallo et al. (2016)	Cognitive (Memory: ↑↑↑↓ Executive Function: ↑)
Chae et al. (2024)	Cognitive (Global Cognition: ↑) Well-Being (↑ 一) Functional (↑↑↑)
Chandler et al. (2019)	Well-Being (一 ↑) Functional (一)
Chantanachai et al. (2024)	Cognitive (Global cognition:一Memory: 一 Executive Function: 一 Speed:一) Functional (一一一一一一一一) Well-Being (一一一)
Choi et al. (2025)	Cognitive (Executive Function: Gerontology Functional Assessment Tool ↑↑ 一 一 Memory: 一) Well-Being (一)
Christogianni et al. (2022)	Cognitive (Sensorimotor: 一 Memory: 一 Speed: 一)
Çinar et al. (2020)	Cognitive (Global Cognition: ↑ Motor:↑ Memory/learning:↑↑↑Visuospatial: ↑ Speed: 一) Functional (↑)
Combourieu Donnezan et al. (2018)	Cognitive (Executive Function ↑) Functional (↑)
Cruz et al. (2013)	Not specified; other (high levels of motivation to use at home)
Danesin et al. (2025)	Cognitive (Memory/Learning: ↑↑一一 Executive Function:↑一一一 Attention:一 Visuosptaial: 一 Language: 一) Well-Being (↑)
De Luca et al. (2016)	Cognitive (Global Cognition: ↑ Executive Function: ↑ ↑ ↑ Attention: ↑) Functional (↑ ↑) Well-Being (↑ ↑)
Dethlefs et al. (2017)	Not specified: other (high enjoyment of using system)
Diaz Baquero et al. (2022)	Cognitive (Global Cognition: ↑ ↑ Executive Function: ↑ ↑ ↑ ↑ ↑ ↑ ↑ ↑ ↑ ↑ ↓一 Working Memory: ↑ ↑ Memory: ↓ Speed: ↑ ↓ Reasoning: ↑) Well-Being (↑)
Djabelkhir-Jemmi et al. (2018)	Cognitive (Global Cognition: ↑ Executive Function: ↑ 一 Memory: ↑ ↑↑ ↑ 一 Working Memory: ↑ Attention/ Speed: ↑ ↑↑ Visuospatial: ↑ ↑ ↑) Well-Being (↑ ↑ 一 一 一 一)
Duff et al. (2022)	Cognitive (Global Cognition ↑, Auditory Memory and Attention: ↑) Functional (↓)
Ferreira et al. (2023)	Cognitive (Global Cognition: 一一 Executive Function: ↑↑一一一一 Speed:一) Well-Being (↑)
Fiatarone Singh et al. (2014)	Cognitive (Global Cognition: ↑ Executive Function: ↑ ↑ ↓ 一 Memory: ↑ ↑ 一一 Attention/Speed: ↑) Functional (↑)
Finn et al. (2014)	Cognitive (Executive Function: ↑ Attention: ↑) Well-Being (↑↑)
Fiorini et al. (2019)	Other
Gaitán et al. (2013)	Cognitive (Global Cognition: ↓ Attention/Speed: ↑ Working Memory: ↑ Memory: ↑ Executive Function: ↓ Orientation: ↓Gnosis: ↑ Decision-Making: ↓) Well-Being (↑ ↑ 一)
Gandelman-Marton et al. (2017)	Cognitive (MMSE: ↑, ADAS-Cog: ↑)
Gigler et al. (2013)	Cognitive (Speed: ↑ Attention: 一 Working memory: 一 Visuo-motor: 一 Executive function: 一 Memory: 一) Functional: (一一) Well-Being (一)
Givon Schaham et al. (2024)	Cognitive (Global Cognition: ↑ Attention: 一一 Memory: 一一一 Executive Function: 一一) Well-Being (一)
Gonzalez et al. (2021)	Cognitive: (Global Cognition: ↑ Working memory: ↑ Attention/Speed: ↑ Memory: ↑↑↑)
Gonzalez-Palau et al. (2014)	Cognitive (Global Cognition: ↑ Memory: ↑↑) Well-Being (↑)
Graessel et al. (2024)	Cognitive (Global cognition: ↑)

Study	Outcome Summary
Hagovská et al. (2017)	Cognitive (Attention: ↑) Well-Being (↑)
Han et al. (2017)	Cognitive (Memory: ↑)
Han et al. (2020)	Cognitive (Global Cognition: ↑ ↑)
Han et al. (2024)	Cognitive (Global cognition: ↑ Memory: ㅡ) Well-Being (ㅡㅡㅡ)
Hang et al. (2019)	Not Specified; Other
Harvey et al. (2020)	Functional (↑)
Harvey et al. (2024)	Cognitive (Global Cognition ㅡ) Functional (ㅡ)
Hassandra et al. (2021)	Not Specified; Other
Hird et al. (2024)	Not specified; other (high levels of engagement)
Hoel et al. (2022)	Well-Being (ㅡ)
Hung et al., (2021)	Not Specified; Other
Hwang et al. (2023)	Cognitive (↑ Attention ㅡ Executive Function: ↑ , Visospatial: ↑ Reasoning: ↑ Memory: ↑) Functional (ㅡ ㅡ ↑ ↑)
Hyer et al. (2016)	Cognitive (Working Memory: ↑) Functional (↑)
Jeong et al. (2025)	Cognitive (Global Cognition: ↑ㅡ Memory: ↑ㅡㅡㅡㅡㅡㅡㅡ Working Memory: ㅡㅡ Attention: ㅡ Language: ㅡ Executive Function: ㅡ Visuospatial: ㅡ Claculation: ㅡ)
Kanaan et al. (2014)	Cognitive (Global Cognition: ↑ Working Memory: ↑ ↑ ↑ ↑ ↑ ↑ Attention: ↑ ↑↑ ↑↑ ↑ ↑ ↑ ↑ ↑ ↑ ↑↑↑↑ Speed ↑) Well-Being (ㅡ)
Kang et al. (2024)	Cognitive (Global Cognition: ㅡㅡ Memory: ↑ Executive Function: ㅡㅡㅡㅡㅡ Visuospatial: ㅡㅡ Language: ㅡㅡㅡ Attention: ㅡ) Well-Being (ㅡㅡㅡ)
Karssemeijer et al. (2019)	Functional (↑ㅡㅡㅡㅡㅡㅡㅡ)
Kim et al. (2020)	Cognitive (Memory: ↑ ↑ Attention: ↑ Visuomotor: ↑ Global Cognition: ↑)
Kim et al. (2021)	Cognitive (Global Cognition ↑)
Latella et al., (2024)	Cognitive (Global cognition: ↑ Executive Functions: ↑ Visuospatial: ↑ Memory: ↑ Intelligence: ↑) Functional (ㅡ)
Lau et al. (2024)	Cognitive (Global cognition:↑ Executive Function:↑ Memory: ↑ Attention: ↑) Functional (↑)
Lee et al. (2018)	Cognitive (Global Congition: ↑↑ Memory: ↑↑ Learning: ↑)
Lee et al. (2020)	Cognitive (Working Memory: ↑ Memory/Learning: ㅡ Attention: ㅡ) Well-Being (↑ㅡ)
Lee et al. (2023)	Cognitive (Global Cognition ㅡ Memory ↑↑↑↑ㅡㅡㅡ Executive Function ↑ㅡ)
Li et al. (2023)	Cognitive (Global Cognition: ↑↑↑ Memory: ㅡ ↑ Attention: ↑↑ Reasoning: ㅡ Language: ↑ Visuospatial: ↑ Executive Function: ↑)
Liang et al. (2017)	Cognitive (Global cognition:ㅡ) Functional (↑ㅡㅡㅡ) Well-Being (↑ㅡㅡㅡㅡㅡㅡㅡㅡㅡ)
Liao et al. (2019)	Cognitive (Global Cognition:↑ Memory:↑↑ Exectutive Function:↑) Functional (↑)
Lin et al. (2021)	Cognitive (Memory: ㅡ Executive Function: ↑)
Lin et al. (2022)	Cognitive (Global cognition: ↑) Functional (↓ ↑ ↑ ↑ ↑ ↑) Well-Being (↑ ↑ ↑)
Lissek et al. (2024)	Cognitive (Global Cognition: ↑ Attention: ↑ ↑ㅡ ㅡ ㅡ ㅡ Memory: ㅡ ㅡ ㅡ ↑ㅡ ㅡ↑ Executive Function: ㅡ ㅡ) Well-Being (↑)
Maeng et al. (2021)	Cognitive (Executive Function: ㅡ↑, Language: ㅡ, Memory: ㅡㅡ↑↑↑ Visuospatial: ↑↑ Speed: ↑ Attention: ↑) Well-Being (ㅡㅡ)
Manca et al. (2021)	Cognitive (Memory: ↑↑↑↓ Attention/Speed: ↑)
Manenti et al. (2020)	Cognitive (Memory: ↑ㅡㅡㅡㅡㅡㅡㅡ Speed: ↑ Visuospatial: ↑ Language: ↑ㅡㅡㅡ Executive Function: ㅡ) Functional (ㅡㅡ) Well-Being (ㅡ)
Manenti et al. (2024)	Cognitive (Global Cognition: ㅡ Memory: ↑ㅡㅡㅡㅡ Intelligence: ㅡ Visuospatial: ㅡ Language: ㅡㅡ Executive Function: ㅡ Speed: ㅡ) Functional (ㅡㅡ) Well-Being (ㅡㅡㅡ)
Manser et al. (2024)	Cognitive (Global cognition ↑; Memory ↑↑———; Attention ————; Executive functions —————; Visuospatial ——; Motor ———————) | Well-Being (— — — —) | Functional (—)
Marin et al. (2022)	Cognitive (Global Cognition: ↑↑ Memory: ↑ Attention: ↑)
Mendoza Laiz et al. (2018)	Cognitive (Attention: ↑ Perception: ↑ Motor: ↑ Language: ↑↑ Memory: ↑↓ ↑)
Mondellini et al. (2022)	Not specificed; other (high acceptance)
Montero-Odasso et al. (2023)	Cognitive (Global Cognition ↑ Memory: ㅡㅡ ㅡㅡVisuospatial: ㅡㅡ Speed:↑ Executive Function:ㅡㅡㅡ Language: ㅡㅡㅡㅡ↑)
Na et al. (2018)	Cognitive (Global cognition:ㅡ Attention/Working Memory: ↑↑ Memory: ↑ Executive Function: ↑) Functional (ㅡ)

Study	Outcome Summary
Na et al. (2025)	Cognitive (Global cognition: ↑ Memory: ↑ Attention: — Executive functions: — Visuospatial: — Language: —) Well-Being (— — — —) Functional (—)
Navarro et al. (2018)	Not specified; Other
Nieto-Vieites et al. (2024)	Cognitive (Executive Function: 一, Memory: 一)
O´Sullivan et al. (2022)	Well-Being (一 一 一)
Oh et al. (2018)	Cognitive (Global cognition: ↑ Attention: ↑ Memory:↑ Visuotemporal: ↑)
Oliveira et al. (2021)	Cognitive (Executive Function: 一一 Global Cognition:↑一) Well-Being (一) Functional (一一)
Park (2022)	Cognitive (Visuospatial: ↑ Memory: ↑)
Park et al. (2019)	Cognitive (Memory:一 一一Language: 一一Visuospatial/Working Memory: ↑ ↑ Attention/Executive Function:一一)
Park (2022)	Cognitive (Visuospatial: ↑ Memory:↑) Other: Prefrontal cortex activity (↑)
Pino et al. (2012)	Not specified; Other
Prinz et al. (2025)	Cognitive (Global Cognition: 一 Speed:一) Functional (↑一一一一) Well-Being (一一)
Rai et al. (2021)	Cognitive (Global Cognition: 一) Well-Being (一一 一一) Functional (一)
Robert et al. (2020)	Cognitive (Global Cognition: 一一 Memory: 一 Attention: 一一一一↑↓一 Executive Function:一) Well-Being (一一 ↓)
Rouse et al. (2019)	Cognitive (Global cognition: ↑ 一 Memory: ↑一 Visuospatial: ↑ 一) Well-Being (↑↓↑ ↑)
Savulich et al. (2017)	Cognitive (Memory: ↑ Learning:↑ Visospatial: ↑ Speed: 一 Global Cognition: 一) Well-Being (一一)
Senczyszyn et al. (2021)	Cognitive (Executive Function: ↑一 Working Memory: 一 Memory:↑ ↑一一一Language: ↑一 Speed:↑ Visuospatial: ↑一) Well-Being (↑)
Senczyszyn et al. (2023)	Cognitive (Global Cognition: ↑ Executive Function: 一 Motor: 一 Speed: 一 Memory: 一↑↑ Learning: ↑)
Shandera-Ochsner et al. (2021)	Functional (↑)
Shin et al. (2020)	Cognitive (Global Cognition: ↑ Attention: ↑ ↑一 一 一 一 Memory: 一 一 一 ↑一 一↑ Executive Function: 一 一) Well-Being (↑)
Shyu et al. (2022)	Cognitive (Global Cognition: 一 一 Memory:↑) Well-Being (↑↑一)
Tan et al., (2025)	Well-Being (↑↑)
Tsiakari et al. (2025)	Cognitive (Global Cognition: 一)
van Santen et al., (2020)	Cognitive (Global cognition:↑ Executive Function: ↑ Speed: ↑) Functional (↑一) Well-Being (↑↑)
Wiloth et al. (2018)	Cognitive (Attention: ↑ ↑ ↑ Motor: ↑ ↑).
Wu et al. (2020)	Not specified; Other
Yang et al. (2022)	Cognitive (Global cognition: ↑↑ Speed: ↑)
Yu et al. (2015)	Cognitive (Global Cognition: 一 Attention: 一 Language: ↑) Well-Being (↑ ↑ 一)
Yu et al. (2025)	Cognitive (Global Cognition: ↑)
Yun et al. (2020)	Not specified; other (positive satisfaction, usability ratings)
Zaccarelli et al. (2013)	Cognitive (Global cognition: ↑ Memory: ↑ Executive Function: ↑ Language: 一) Functional (↑)
Zając-Lamparska et al. (2019)	Cognitive (Global Cognition: 一一 Language: 一 一 Memory: 一 一 一一 Attention: 一一 ↑ Visuospatial: ↑)
Zhang et al. (2025)	Cognitive (Global cognition: 一 Memory: ↑↑↑ Learning:↑ Language: ↑↑ Attention: ↑ Executive Function: ↑↑) Functional (↑)
Zhu et al. (2022)	Cognitive (Executive Function: ↑ Attention: ↑ Memory: ↑ Global Cognition: ↑) Well being (↑↑).
Zuschnegg et al (2025)	Cognitive (Global Cognition: ↑ ─ Memory/Learning:一一一一一 Attention: 一 Working Memory: 一 Executive Function: 一一一一一 Speed: 一一)
