# Supplementary material for: The use of digital technology in non-pharmacological cognitive and psychosocial interventions for people with dementia and mild cognitive impairment: A scoping review
Source: PLoS One. 2026 Apr 30;21(4):e0346008. doi: 10.1371/journal.pone.0346008 (PMC13132441; doi:10.1371/journal.pone.0346008)
Supplement: S4 Supplementary Material — (PDF) [file pone.0346008.s004.pdf]

S2: Supplementary Material - Search Strategy

First iteration of search for terms:

N-gram (1-2 word) frequency search based on TERA (https://tera-tools.com/word-freq) word frequency search algorithm using Scopus database search output for the initial seed string:

(“dementia” OR “Alzheimer*” OR "MCI" OR "mild cognitive impairment") AND (“cognitive training” OR "cognitive intervention" OR “psychosocial intervention*” OR “cognitive stimulation”) AND (digi* OR computer* OR web* OR technolog*)

word	unique	title	abstract	keywords	points
cognitive	1115	1148	6055	3958	11161
training	831	436	2521	921	3878
dementia	830	294	1776	1131	3201
study	826	198	1244	929	2371
cognitive training	759	331	1330	614	2275
intervention	686	127	1616	344	2087
impairment	716	342	940	794	2076
cognitive impairment	691	336	881	777	1994
memory	529	79	938	793	1810
mild	662	316	787	702	1805
disease	573	151	535	957	1643
therapy	553	64	419	1133	1616
interventions	548	135	1350	118	1603
aged	591	10	140	1446	1596
cognition	605	57	533	967	1557
mild cognitive	584	269	588	691	1548
older	474	263	1092	114	1469
controlled	573	187	373	895	1455
group	464	19	1364	34	1417
function	507	87	842	376	1305
review	392	250	564	485	1299
based	594	185	1026	68	1279
adults	436	242	940	96	1278
mci	360	33	1166	59	1258
patients	428	146	1091	14	1251
©	1103	0	1225	0	1225
trial	468	185	478	553	1216
studies	498	9	1068	116	1193
clinical	585	49	457	665	1171
health	466	42	547	554	1143
results	780	19	1111	6	1136
people	423	163	928	35	1126

quality	410	19	679	412	1110
randomized	483	175	422	512	1109
assessment	526	25	396	686	1107
older adults	388	207	795	92	1094
care	405	58	595	436	1089
stimulation	377	135	543	395	1073
life	472	28	507	527	1062
test	386	2	409	621	1032
participants	474	2	1029	0	1031
analysis	426	118	508	384	1010
human	867	4	68	920	992
treatment	433	34	492	391	917
randomized controlled	454	146	236	507	889
rehabilitation	373	53	354	461	868
exercise	238	55	440	366	861
brain	293	68	391	379	838
effects	436	117	701	13	831
systematic	288	187	289	289	765
using	487	53	706	0	759
outcome	382	1	282	475	758
physical	283	48	495	209	752
technology	346	59	439	248	746
control	397	13	628	100	741
social	273	31	418	288	737
female	446	1	33	698	732
mental	401	17	253	448	718
controlled trial	404	133	133	447	713
methods	604	12	674	18	704
male	440	1	8	691	700
computer	372	57	340	292	689
research	410	23	530	135	688
elderly	354	59	328	285	672
cognitive function	323	65	522	64	651
risk	278	28	435	178	641
activity	340	26	296	317	639
virtual	181	90	251	290	631
reality	182	85	221	320	626
article	482	0	74	548	622
effect	327	47	486	89	622
cognitive stimulation	276	92	370	153	615
performance	322	25	382	208	615
computerized	274	110	362	141	613
attention	272	17	358	224	599

humans	590	0	9	588	597
significant	396	0	592	3	595
systematic review	259	175	170	248	593
evidence	296	17	536	33	586
executive	254	16	303	264	583
decline	310	49	454	80	583
functional	236	34	327	221	582
data	348	10	486	83	579
daily	284	14	316	248	578
activities	288	20	429	111	560
cognition cognitive	387	0	3	555	558
design	311	40	357	154	551
included	343	0	541	0	541
neuropsychological	261	6	196	337	539
effectiveness	327	70	338	131	539
cct	110	2	530	6	538
outcomes	318	28	493	11	532
depression	208	14	213	296	523
after	324	24	496	0	520
living	257	33	313	173	519
program	233	49	385	82	516
virtual reality	155	72	167	271	510
all	384	0	506	0	506
system	242	36	341	123	500
scale	248	0	195	305	500
meta	172	102	266	130	498
between	346	11	486	0	497
support	264	27	380	87	494
improve	382	38	456	0	494
groups	293	3	471	14	488
cognitive decline	269	44	358	77	479
cognitive rehabilitation	279	28	140	304	472
alzheimers	229	73	253	138	464
caregivers	146	37	341	86	464
digital	169	60	280	123	463
alzheimer	281	14	44	404	462
patient	268	11	173	278	462
trials	251	30	358	73	461
individuals	255	35	421	4	460
non	256	50	389	20	459
computerized cognitive	220	85	251	122	458
defect	445	1	1	448	450
ad	137	5	434	6	445

cognitive defect	444	0	0	444	444
learning	191	13	205	218	436
psychosocial	190	37	231	166	434
aging	239	20	192	219	431
home	186	41	262	108	411
executive function	216	8	154	248	410
behavioral	200	19	142	241	402
dysfunction	354	8	36	357	401
background	398	0	400	1	401
alzheimer disease	248	10	22	368	400
middle	217	4	26	367	397
efficacy	260	46	329	20	395
effective	292	5	384	2	391
functions	236	22	315	54	391
age	243	10	324	51	385
time	235	6	304	72	382
alzheimers disease	202	62	189	131	382
controlled study	363	12	11	359	382
meta analysis	146	90	161	130	381
potential	291	9	361	11	381
showed	282	0	380	0	380
middle aged	206	4	14	359	377
cognitive dysfunction	336	7	18	350	375
related	263	22	337	15	374
both	270	0	373	0	373
ci	113	0	372	0	372
however	316	0	368	0	368
state	271	5	165	196	366
games	143	25	229	104	358
improvement	243	19	326	8	353
vr	75	12	328	12	352
adult	277	1	45	305	351
working	180	18	177	152	347
conducted	290	0	346	0	346
game	130	37	214	93	344
impairment mci	315	10	316	15	341
most	251	1	339	0	340
follow	195	2	212	122	336
weeks	201	2	334	0	336
topic	202	0	19	315	334
through	249	22	311	0	333
control group	203	2	326	4	332
combined	162	43	259	27	329

web	244	15	269	43	327
conclusions	314	0	324	0	324
including	259	4	318	0	322
healthy	176	31	239	51	321
measures	204	5	307	8	320
task	173	15	212	92	319
years	232	6	309	3	318
baseline	202	9	303	3	315
changes	195	9	303	3	315
tests	226	3	165	146	314
working memory	157	18	145	151	314
objective	274	1	311	2	314
self	143	9	207	98	314
compared	224	3	309	0	312
disorders	186	18	140	154	312
feasibility	122	46	169	97	312
months	153	2	308	0	310
interaction	160	18	159	131	308
information	177	12	186	109	307
week	202	8	299	0	307
primary	203	9	247	47	303
global	182	7	271	23	301
findings	255	7	293	1	301
current	172	21	198	81	300
examination	225	1	93	205	299
found	222	0	299	0	299
psychology	275	0	18	277	295
s	270	0	293	1	294
imaging	123	3	46	244	293
symptoms	170	17	251	24	292
science	231	0	252	39	291
magnetic	127	8	48	233	289
sessions	201	1	284	2	287
author	274	0	284	0	284
among	202	30	254	0	284
term	190	13	211	59	283
improved	212	3	275	0	278
high	202	4	248	25	277
alzheimer’s	130	53	147	75	275
post	175	13	251	10	274
very	229	0	87	186	273
caregiver	136	8	107	157	272
mini	213	2	92	178	272

technologies	170	25	202	45	272
functioning	161	17	236	18	271
major	240	4	63	204	271
improving	209	10	260	0	270
other	221	4	264	1	269
language	133	9	151	108	268
development	189	30	202	36	268
application	161	24	192	51	267
improvements	189	4	261	0	265
assessed	221	0	263	0	263
network	97	24	117	121	262
cognitive therapy	194	2	7	251	260
verbal	137	1	124	134	259
status	162	4	123	131	258
lifestyle	84	17	152	87	256
population	191	9	208	38	255
daily living	150	5	147	102	254
treatment outcome	144	0	2	251	253
conclusion	248	0	253	0	253
mini mental	202	0	77	175	252
processing	129	8	137	106	251
method	205	13	174	64	251
author s	249	0	250	0	250
mobile	103	27	123	99	249
state examination	197	0	69	178	247
mental state	197	0	71	176	247
based cognitive	134	59	174	13	246
significantly	181	0	246	0	246
factors	155	9	184	53	246
pharmacological	147	26	214	5	245
pilot	114	58	88	98	244
physical activity	125	10	106	126	242
user	110	17	170	55	242
procedures	233	0	23	218	241
total	204	1	233	6	240
positive	187	2	228	10	240
community	133	25	161	53	239
anxiety	108	3	106	129	238
disorder	144	10	61	167	238
scores	154	1	230	2	233
evaluation	163	21	156	56	233
early	147	19	178	35	232
controlled trials	164	20	149	63	232

benefits	187	4	224	4	232
during	165	17	214	1	232
provide	192	2	229	0	231
published	204	1	229	0	230
health care	147	5	47	178	230
medical	145	2	125	102	229
alzheimer’s disease	119	45	115	67	227
cognitive assessment	164	3	104	120	227
future	204	12	211	4	227
low	127	5	209	12	226
tool	178	12	194	19	225
education	142	2	127	95	224
approach	169	13	198	13	224
while	196	1	223	0	224
specific	169	1	221	1	223
authors	196	0	222	0	222
protocol	129	72	107	42	221
music	58	14	124	82	220
long	151	12	183	25	220
associated	160	11	203	6	220
impact	163	23	193	4	220
behavior	136	7	71	141	219
ct	44	1	218	0	219
different	175	10	207	2	219
active	133	10	195	14	219
analyses	149	7	209	1	217
transcranial	60	26	56	134	216
further	195	1	213	0	214
tasks	133	4	209	1	214
cognitive intervention	105	39	126	48	213
programs	123	12	189	11	212
reported	156	3	207	2	212
mean	140	0	210	2	212
prevention	128	22	111	79	212
speed	98	8	143	59	210
those	158	1	208	0	209
cognitive functions	138	14	155	40	209
literature	155	12	180	14	206
resonance	109	1	29	175	205
practice	127	13	107	85	205
new	143	9	183	12	204
moderate	134	19	181	4	204
magnetic resonance	108	1	27	175	203

cognitive interventions	97	17	159	26	202
communication	107	10	114	77	201
cognitive behavioral	109	2	15	184	201
domains	148	4	194	3	201
performed	168	1	199	0	200
e	126	4	174	21	199
systems	115	5	96	97	198
number	156	0	194	4	198
motor	94	9	134	54	197
resonance imaging	107	1	26	170	197
score	128	0	181	16	197
criteria	144	1	193	3	197
aimed	181	2	194	0	196
usability	72	16	143	36	195
single	135	8	118	69	195
level	139	2	170	23	195
being	150	11	179	4	194
behavioral therapy	106	1	9	183	193
each	157	0	192	0	192
applications	124	14	130	48	192
identified	147	0	192	0	192
psychological	114	8	108	75	191
overall	150	1	189	1	191
assess	162	5	184	1	190
neurodegenerative	141	6	80	103	189
assisted	153	10	41	138	189
differences	143	2	184	3	189
engagement	114	10	155	24	189
robot	52	22	109	57	188
computer based	93	33	146	8	187
stroke	47	19	102	66	187
smd	50	0	186	0	186
questionnaire	127	0	57	129	186
very elderly	186	0	0	186	186
visual	111	5	100	81	186
clinical trial	139	23	66	95	184
female humans	184	0	0	184	184
evaluate	166	1	183	0	184
clinical study	183	1	0	182	183
major clinical	183	0	1	182	183
paper	146	3	175	5	183
serious	69	20	116	47	183
mood	91	8	134	41	183

diseases	145	7	67	109	183
cochrane	133	0	163	19	182
databases	167	0	178	4	182
robots	51	12	94	75	181
change	110	8	142	31	181
online	85	25	121	34	180
daily life	165	3	38	138	179
assistive	68	23	80	76	179
experimental	118	6	156	17	179
making	125	3	100	75	178
long term	125	9	146	21	176
aim	166	0	175	0	175
non pharmacological	114	24	150	1	175
diagnosis	110	9	111	55	175
evaluated	152	0	175	0	175
rights	173	0	173	1	174
impairments	118	13	101	59	173
skills	98	16	137	20	173
experience	114	13	132	27	172
developed	148	3	169	0	172
management	97	18	120	34	172
blind	113	13	61	97	171
montreal	130	0	62	109	171
reserved	170	0	171	0	171
search	134	2	162	6	170
model	115	10	129	30	169
domain	113	10	154	5	169
secondary	131	6	147	16	169
strategies	117	11	149	9	169
need	141	2	163	3	168
reviews	99	7	119	42	168
personalized	101	10	117	41	168
present	145	1	167	0	168
montreal cognitive	130	0	61	107	168
defect cognitive	167	0	0	167	167
all rights	166	0	167	0	167
nursing	70	11	70	85	166
tdcs	25	3	156	6	165
pubmed	164	0	165	0	165
study male	165	0	0	165	165
aged aged	164	0	0	165	165
rights reserved	163	0	164	0	164
therapy cognitive	109	0	7	156	163

humans male	162	0	0	162	162
neuropsychological tests	137	0	46	116	162
factor	116	5	45	111	161
qualitative	80	17	89	55	161
increased	126	0	160	0	160
trial topic	150	0	0	160	160
without	119	21	138	0	159
outcome assessment	144	0	2	157	159
limited	150	0	159	0	159
aged article	159	0	0	159	159
aims	145	0	159	0	159
aerobic	57	10	95	53	158
medicine	102	4	53	99	156
adherence	76	8	136	10	154
first	127	3	151	0	154
carers	43	15	130	7	152
mental health	84	2	67	83	152
across	122	2	146	4	152
therapeutic	96	3	132	17	152
mixed	111	16	117	18	151
completed	114	0	151	0	151
cost	73	6	107	37	150
exercises	90	4	142	4	150
psychosocial interventions	85	17	99	34	150
implementation	86	11	128	11	150
reminiscence	55	20	90	39	149
embase	132	0	126	23	149
rating	100	0	52	97	149
ability	114	2	121	26	149
multiple	104	8	118	23	149
neuropsychological test	135	0	20	128	148
month	91	8	140	0	148
video	76	6	61	81	148
received	113	0	148	0	148
stimulation therapy	68	27	70	50	147
abilities	115	6	138	3	147
due	124	4	143	0	147
geriatric	111	4	62	80	146
about	116	3	143	0	146
whether	121	0	146	0	146
bias	81	0	132	13	145
multi	85	19	123	3	145
software	94	1	87	56	144

pilot study	88	36	45	63	144
various	125	0	144	0	144
person	94	9	98	37	144
direct	64	17	56	71	144
difference	107	0	131	13	144
subjective	77	19	97	27	143
intervention group	80	0	143	0	143
designed	125	5	138	0	143
cst	31	2	134	6	142
inclusion	111	4	134	4	142
short	109	3	77	61	141
association	122	5	108	28	141
g	76	0	141	0	141
male middle	109	0	0	141	141
computer assisted	128	6	16	119	141
important	128	0	141	0	141
older people	72	26	104	10	140
journal	132	0	40	100	140
persons	86	21	110	9	140
objectives	137	0	140	0	140
stress	62	6	74	60	140
approaches	100	7	130	3	140
digit	97	0	91	49	140
before	128	0	139	0	139
female human	139	0	0	139	139
monitoring	81	9	83	47	139
needs	98	4	128	6	138
life activity	138	0	0	138	138
suggest	125	0	138	0	138
healthcare	85	10	108	20	138
articles	89	0	137	0	137
psychosocial intervention	99	6	42	89	137
cognitive impairments	93	9	70	58	137
platform	77	16	114	7	137
investigate	129	1	136	0	137
cortex	57	2	41	93	136
devices	89	5	93	38	136
enhance	121	12	123	1	136
family	64	11	96	28	135
male memory	113	0	0	135	135
medline	118	0	96	39	135
plasticity	67	4	41	90	135
rcts	63	0	133	1	134

size	97	0	91	43	134
aged cognition	104	0	0	133	133
needed	128	1	132	0	133
demonstrated	117	0	133	0	133
better	109	0	132	0	132
multidomain	47	16	97	19	132
procedure	122	0	13	119	132
assessments	107	0	123	8	131
pd	28	1	128	2	131
following	116	5	126	0	131
benefit	106	1	120	10	131
episodic	59	4	55	71	130
case	72	22	72	36	130
burden	70	4	96	30	130
searched	121	0	130	0	130
intelligence	85	7	34	89	130
registration	116	0	129	1	130
general	84	1	109	20	130
individual	98	8	116	5	129
processing speed	67	3	80	46	129
pre	103	3	126	0	129
help	112	4	115	10	129
how	96	7	121	0	128
increase	116	0	127	1	128
duration	97	0	73	55	128
adult aged	124	0	0	128	128
sample	102	1	112	15	128
identify	115	1	126	0	127
cognitive functioning	79	11	113	3	127
physical exercise	69	14	97	16	127
current stimulation	43	17	34	75	126
spatial	60	6	69	50	125
multimodal	58	18	76	31	125
neurodegenerative diseases	112	4	23	98	125
condition	79	2	113	9	124
report	86	15	65	44	124
within	108	2	122	0	124
conditions	94	3	116	5	124

Second iteration of search for terms:

N-gram (1-2 word) frequency search based on TERA (https://tera-tools.com/word-freq) word frequency search algorithm using Scopus database search output for the seed string:

(“dementia” OR “Alzheimer*” OR "MCI" OR "mild cognitive impairment") AND (“cognitive training” OR "cognitive intervention" OR “psychosocial intervention*” OR “cognitive stimulation”) AND (digi* OR computer* OR web* OR technolog* OR robot* OR *game OR gam* OR vr OR "virtual reality" OR "computeri#ed cognitive" OR cct)

word	unique	title	abstract	keywords	points
cognitive	1330	1341	7064	4599	13004
training	992	506	2951	1093	4550
dementia	977	353	2105	1342	3800
study	973	234	1449	1089	2772
cognitive training	908	378	1552	729	2659
impairment	843	404	1086	938	2428
intervention	795	152	1869	397	2418
cognitive impairment	815	397	1022	919	2338
mild	782	371	921	819	2111
memory	624	94	1068	884	2046
disease	670	176	625	1131	1932
therapy	644	75	496	1288	1859
aged	677	12	160	1647	1819
mild cognitive	691	320	689	808	1817
cognition	708	70	627	1107	1804
older	577	314	1331	154	1799
interventions	629	152	1504	129	1785
controlled	674	218	440	1052	1710
group	547	20	1625	36	1681
adults	530	286	1155	130	1571
function	607	103	984	460	1547
based	695	227	1201	76	1504
patients	517	179	1283	20	1482
mci	426	39	1356	74	1469
©	1302	0	1446	0	1446
trial	552	216	564	658	1438
review	436	275	621	522	1418
studies	571	9	1213	133	1355
older adults	472	247	978	125	1350
randomized	566	212	504	608	1324
clinical	671	54	508	761	1323
people	503	194	1079	45	1318
results	913	20	1287	6	1313

health	534	50	627	606	1283
stimulation	453	152	631	463	1246
assessment	603	29	449	764	1242
quality	473	20	752	459	1231
care	459	63	683	484	1230
participants	562	2	1208	0	1210
life	543	30	582	591	1203
human	1005	8	103	1064	1175
analysis	503	133	580	440	1153
test	444	2	473	676	1151
randomized controlled	531	172	283	600	1055
treatment	485	40	576	435	1051
exercise	295	67	528	451	1046
rehabilitation	435	64	430	540	1034
brain	345	77	459	447	983
effects	516	140	812	23	975
physical	341	65	610	254	929
control	473	16	759	122	897
using	573	70	826	0	896
social	329	40	500	355	895
controlled trial	476	157	168	528	853
virtual	244	131	316	403	850
female	511	1	37	801	839
systematic	314	209	316	309	834
mental	464	20	294	518	832
outcome	426	1	314	516	831
reality	245	123	276	430	829
elderly	430	76	392	344	812
male	505	2	11	797	810
methods	693	16	774	19	809
research	476	23	607	150	780
activity	406	30	357	372	759
technology	350	59	446	250	755
cognitive function	380	71	603	76	750
risk	323	33	500	200	733
performance	382	28	452	251	731
executive	316	24	379	326	729
effect	392	60	553	110	723
article	553	0	85	628	713
cognitive stimulation	335	105	428	179	712
attention	333	21	419	272	712
virtual reality	218	110	219	379	708
significant	464	0	702	3	705

decline	374	58	544	96	698
computer	374	57	341	293	691
design	369	51	437	195	683
daily	335	16	377	287	680
humans	669	0	12	664	676
evidence	346	21	610	34	665
games	222	57	438	169	664
activities	331	23	491	132	646
systematic review	284	196	185	264	645
functional	269	36	363	245	644
cognition cognitive	443	0	3	641	644
data	392	10	539	91	640
effectiveness	378	83	394	145	622
neuropsychological	302	8	222	387	617
included	386	0	614	0	614
after	390	25	588	0	613
computerized	274	110	362	141	613
outcomes	363	30	569	11	610
system	285	50	419	138	607
living	295	39	359	204	602
program	269	56	448	94	598
depression	238	14	240	330	584
all	446	0	579	0	579
scale	291	1	238	338	577
groups	343	3	553	16	572
cognitive decline	319	52	425	93	570
between	398	15	553	0	568
improve	441	41	524	0	565
meta	192	114	305	143	562
game	209	56	333	158	547
cct	113	2	537	6	545
cognitive rehabilitation	322	31	161	351	543
alzheimers	260	83	298	161	542
non	303	61	456	24	541
support	298	28	419	90	537
trials	295	32	410	87	529
learning	218	19	247	262	528
alzheimer	321	15	50	460	525
ad	157	5	504	8	517
patient	307	12	191	313	516
individuals	287	43	467	4	514
defect	506	1	1	509	511
aging	286	25	228	253	506

cognitive defect	505	0	0	505	505
executive function	267	13	188	303	504
caregivers	165	39	369	93	501
functions	291	27	401	69	497
vr	104	15	447	16	478
home	211	47	305	118	470
psychosocial	211	39	246	181	466
digital	170	60	280	124	464
background	458	0	461	1	462
dysfunction	403	10	42	408	460
behavioral	236	22	167	271	460
efficacy	300	53	381	25	459
effective	347	5	450	3	458
computerized cognitive	220	85	251	122	458
potential	351	12	431	15	458
alzheimer disease	284	10	26	418	454
both	325	0	450	0	450
time	283	7	353	87	447
middle	242	5	29	413	447
controlled study	425	15	12	419	446
alzheimers disease	227	70	219	154	443
showed	331	0	443	0	443
age	281	11	370	52	433
cognitive dysfunction	381	8	22	395	425
middle aged	230	5	16	404	425
meta analysis	164	102	181	141	424
related	298	26	382	16	424
state	310	5	194	224	423
however	363	0	420	0	420
task	208	19	277	117	413
ci	123	0	412	0	412
weeks	246	2	408	0	410
interaction	216	23	222	163	408
improvement	283	20	380	8	408
control group	242	2	399	4	405
conducted	339	0	403	0	403
feasibility	151	61	217	123	401
through	304	28	372	0	400
impairment mci	369	10	370	18	398
adult	311	1	51	344	396
working	209	19	203	171	393
robot	99	50	227	110	387
week	253	8	379	0	387

follow	219	2	239	139	380
compared	267	5	375	0	380
topic	227	0	24	350	374
healthy	201	37	276	60	373
combined	182	50	294	28	372
most	276	1	368	0	369
objective	319	2	364	2	368
conclusions	356	0	367	0	367
years	271	8	354	3	365
changes	227	11	348	5	364
tests	260	3	188	172	363
including	293	5	357	0	362
sessions	249	1	355	5	361
measures	233	5	345	9	359
among	244	39	320	0	359
baseline	235	10	344	3	357
symptoms	203	24	302	30	356
findings	301	9	344	1	354
working memory	181	19	165	170	354
disorders	208	21	162	166	349
s	319	0	345	3	348
current	205	23	234	90	347
term	226	16	262	69	347
found	262	0	344	0	344
self	160	10	229	105	344
examination	258	1	107	234	342
serious	99	37	221	81	339
primary	230	9	278	48	335
global	206	7	301	25	333
psychology	311	0	20	312	332
months	169	2	329	0	331
high	241	6	293	31	330
improving	242	13	317	0	330
author	320	0	330	0	330
information	195	12	205	112	329
web	244	15	269	43	327
functioning	191	19	285	22	326
very	275	0	99	223	322
alzheimer’s	153	60	174	85	319
language	157	12	180	126	318
development	223	37	235	45	317
magnetic	143	8	54	254	316
improvements	221	6	310	0	316

other	263	4	310	1	315
improved	242	3	311	0	314
imaging	134	3	53	257	313
mini	245	4	106	203	313
post	204	13	286	12	311
during	216	22	285	1	308
pharmacological	179	34	267	6	307
science	245	2	265	39	306
application	186	29	220	57	306
major	273	4	70	231	305
processing	148	11	174	118	303
significantly	220	0	302	0	302
assessed	252	0	301	0	301
user	137	20	213	68	301
verbal	160	1	155	143	299
daily living	170	6	170	121	297
robots	86	22	150	124	296
caregiver	150	9	119	168	296
author s	293	0	294	0	294
positive	229	3	278	12	293
pilot	140	72	108	113	293
community	158	32	196	64	292
mini mental	232	0	90	199	289
conclusion	283	0	289	0	289
total	244	1	280	7	288
network	107	27	129	132	288
cognitive therapy	215	3	9	276	288
state examination	227	0	82	204	286
mental state	227	0	86	200	286
population	215	9	233	44	286
physical activity	147	11	125	147	283
long	190	18	232	33	283
status	182	4	134	145	283
method	232	15	196	72	283
disorder	168	12	77	193	282
based cognitive	153	68	195	15	278
treatment outcome	157	0	2	274	276
approach	212	15	246	14	275
different	210	12	260	2	274
evaluation	187	26	183	65	274
procedures	265	0	25	248	273
technologies	171	25	203	45	273
while	238	1	271	0	272

further	244	1	269	1	271
factors	174	10	206	55	271
behavior	159	10	97	162	269
lifestyle	94	18	159	92	269
provide	226	2	266	0	268
low	151	7	245	16	268
scores	177	1	264	2	267
tasks	162	6	259	2	267
music	77	18	144	104	266
early	168	21	208	37	266
controlled trials	190	22	169	75	266
alzheimer’s disease	141	51	137	77	265
education	161	4	155	106	265
mobile	113	31	129	105	265
prevention	153	27	134	101	262
cognitive assessment	189	4	121	137	262
tool	203	14	223	24	261
cognitive functions	172	16	191	52	259
impact	200	25	230	4	259
engagement	136	17	214	28	259
usability	98	24	187	47	258
anxiety	120	3	113	141	257
associated	191	11	239	7	257
future	231	13	239	5	257
benefits	206	6	243	6	255
speed	118	9	177	66	252
published	222	1	250	0	251
health care	161	6	53	191	250
active	153	13	223	14	250
motor	117	14	166	69	249
specific	191	1	246	2	249
analyses	174	7	239	1	247
performed	204	1	246	0	247
level	173	3	218	26	247
authors	219	0	246	0	246
medical	159	2	134	109	245
transcranial	72	28	64	150	242
those	185	1	241	0	242
protocol	146	83	115	44	242
neurodegenerative	183	8	94	139	241
mean	159	0	238	3	241
diseases	191	8	89	143	240
reported	175	3	234	2	239

moderate	162	20	215	4	239
new	170	10	215	13	238
ct	48	1	237	0	238
score	151	0	219	18	237
cognitive intervention	121	46	137	53	236
programs	137	13	209	13	235
experimental	146	6	208	20	234
systems	135	5	104	122	231
practice	146	13	126	92	231
being	171	11	210	5	226
long term	155	12	186	28	226
serious games	67	20	140	66	226
model	140	14	167	45	226
single	158	9	143	74	226
aimed	210	2	223	0	225
e	147	4	198	22	224
paper	180	3	215	5	223
very elderly	223	0	0	223	223
assistive	85	26	95	102	223
cognitive behavioral	120	2	16	204	222
domains	165	4	215	3	222
resonance	120	1	33	187	221
visual	129	5	124	92	221
non pharmacological	140	31	187	2	220
number	174	0	216	4	220
evaluate	198	1	219	0	220
video	109	10	84	125	219
literature	166	13	190	15	218
magnetic resonance	118	1	30	187	218
each	181	0	217	0	217
developed	183	6	209	2	217
communication	116	10	120	85	215
questionnaire	148	0	67	148	215
making	139	6	121	88	215
differences	163	2	207	5	214
criteria	157	1	208	5	214
cognitive interventions	107	19	164	30	213
experience	141	16	164	33	213
clinical trial	162	24	74	115	213
female humans	213	0	0	213	213
behavioral therapy	116	1	9	203	213
resonance imaging	117	1	29	182	212
assisted	164	14	56	142	212

assess	184	5	206	1	212
clinical study	210	1	0	209	210
major clinical	210	0	1	209	210
overall	168	1	208	1	210
applications	136	18	141	51	210
evaluated	184	0	210	0	210
smd	55	0	209	0	209
identified	163	0	209	0	209
psychological	127	8	120	80	208
aerobic	72	13	129	66	208
rights	205	0	205	1	206
daily life	188	3	45	156	204
impairments	138	13	118	73	204
montreal	154	1	77	126	204
reserved	203	0	204	0	204
change	125	9	159	34	202
aim	192	0	201	0	201
databases	185	0	197	4	201
present	173	1	199	0	200
montreal cognitive	153	1	75	124	200
stroke	51	19	108	73	200
all rights	198	0	199	0	199
mood	101	8	145	43	196
rights reserved	195	0	196	0	196
increased	155	1	194	0	195
skills	115	17	157	21	195
strategies	133	12	173	9	194
blind	127	16	70	107	193
aged aged	192	0	0	193	193
therapy cognitive	130	0	13	180	193
personalized	117	10	137	45	192
diagnosis	124	9	123	60	192
abilities	147	8	176	7	191
online	90	26	129	36	191
humans male	190	0	0	190	190
secondary	147	7	164	19	190
cochrane	139	0	169	19	188
therapeutic	117	7	160	21	188
computer based	93	33	146	8	187
management	109	19	130	38	187
need	159	3	179	4	186
search	147	2	176	7	185
reviews	108	7	132	46	185

study male	185	0	0	185	185
aged article	185	0	0	185	185
qualitative	93	18	104	63	185
without	141	23	161	0	184
exercises	114	5	175	4	184
limited	172	0	182	1	183
medicine	114	6	69	108	183
factor	133	5	56	121	182
domain	123	11	164	7	182
defect cognitive	182	0	0	182	182
neuropsychological tests	156	0	49	133	182
ability	137	2	149	31	182
trial topic	170	0	0	181	181
nursing	79	11	79	90	180
designed	157	5	174	0	179
pilot study	110	44	58	75	177
subjective	95	23	120	34	177
aims	162	0	177	0	177
mental health	91	5	77	94	176
first	147	3	173	0	176
older people	88	32	128	15	175
pd	39	3	169	2	174
reminiscence	64	23	105	46	174
pubmed	172	0	173	0	173
received	129	1	172	0	173
persons	101	25	133	14	172
outcome assessment	157	0	2	170	172
tdcs	26	3	161	7	171
adherence	88	8	153	10	171
due	145	4	165	0	169
cost	81	8	117	44	169
enhance	151	16	152	1	169
across	134	3	161	4	168
important	152	0	167	1	168
stimulation therapy	77	28	80	59	167
neurodegenerative diseases	151	5	30	132	167
approaches	115	10	154	3	167
neuropsychological test	153	0	22	144	166
person	106	10	107	49	166
multi	94	23	136	5	164
completed	126	0	164	0	164
female human	164	0	0	164	164
intervention group	91	0	164	0	164

whether	136	0	164	0	164
multiple	118	9	132	23	164
month	100	8	155	0	163
cognitive impairments	110	9	83	71	163
short	128	3	95	65	163
geriatric	123	4	68	91	163
suggest	150	0	163	0	163
observed	145	0	161	2	163
levels	117	4	157	2	163
software	108	1	98	63	162
difference	121	0	148	14	162
inf	19	0	162	0	162
investigate	153	1	161	0	162
rating	110	0	54	107	161
mixed	118	17	125	19	161
association	138	7	126	28	161
size	115	0	109	52	161
about	130	3	158	0	161
increase	145	0	159	1	160
healthcare	105	10	122	28	160
needed	154	1	159	0	160
objectives	156	0	159	0	159
various	138	0	159	0	159
before	148	0	159	0	159
male middle	123	0	0	159	159
when	135	1	158	0	159
demonstrated	141	0	159	0	159
journal	149	0	47	111	158
needs	111	4	148	6	158
implementation	92	12	134	12	158
immersive	67	29	104	24	157
bias	91	0	144	13	157
embase	140	0	134	23	157
traditional	100	9	139	9	157
psychosocial interventions	90	17	104	36	157
sample	125	2	137	18	157
episodic	74	5	65	86	156
life activity	156	0	0	156	156
g	88	0	156	0	156
individual	116	9	139	7	155
carers	46	15	133	7	155
plasticity	81	4	46	105	155
general	103	1	133	21	155

better	128	0	154	0	154
aged cognition	120	0	0	154	154
condition	100	2	140	11	153
rcts	72	0	152	1	153
registration	137	0	152	1	153
work	116	2	135	16	153
monitoring	90	9	94	50	153
users	94	3	141	8	152
stress	69	7	78	67	152
psychosocial intervention	113	7	44	100	151
how	112	8	143	0	151
direct	67	18	57	75	150
cognitive functioning	96	12	134	4	150
intelligence	97	7	43	100	150
subjects	89	10	136	4	150
following	132	7	142	0	149
neuropsychiatric	63	10	101	38	149
processing speed	75	4	95	50	149
platform	86	17	125	7	149
conditions	113	4	139	6	149
help	130	5	132	12	149
identify	133	1	147	0	148
higher	123	2	132	14	148
promising	136	2	146	0	148
benefit	118	2	134	12	148
assessments	123	0	135	12	147
male memory	123	0	0	147	147
devices	98	6	98	43	147
inclusion	115	4	138	4	146
pre	115	3	143	0	146
articles	97	0	145	0	145
cst	33	2	137	6	145
physical exercise	78	17	109	18	144
physiology	144	1	0	143	144
multidomain	49	17	106	21	144
procedure	133	0	13	130	143
springer	114	0	142	0	142
