# Supplementary material for: The use of digital technology in non-pharmacological cognitive and psychosocial interventions for people with dementia and mild cognitive impairment: A scoping review
Source: PLoS One. 2026 Apr 30;21(4):e0346008. doi: 10.1371/journal.pone.0346008 (PMC13132441; doi:10.1371/journal.pone.0346008)
Supplement: Attachment — Submitted filename: Peer-Review-Report_PONE-D-25-01664.pdf [file pone.0346008.s005.pdf]

Peer Review Report to Manuscript ‘PONE-D-25-01664’ - The Use of Digital Technology in Non-Pharmacological Cognitive and Psychosocial Interventions for People with Dementia and Mild Cognitive Impairment: A Scoping Review

1 Overview:

I am grateful for the opportunity to review this manuscript, which presents a scoping review on digital health technology-enhanced cognitive and psychosocial interventions in neurocognitive disorders. The review addresses relevant research gaps, which have been well elaborated on in the Introduction. I’d particularly like to commend the authors for committing to completing such a high volume of work. The results are relevant and useful, highlighting that there are still vast opportunities for improvement for unsupervised home-based solutions to optimize healthcare resource allocations in the treatment and management of neurocognitive disorders with the help of digital health technologies.

However, I have concerns that the search strategy might not have been sensitive enough to capture all relevant literature and the methodological choice in the screening of records has resulted in an incomplete picture of the current stage of evidence. The search strategy and screening process are the most critical elements in ensuring the validity of the results of such a review. Therefore, I provide below detailed recommendations for improvements to these and additional aspects of this work to strengthen its completeness, robustness, and future impact in the field.

2 Major Comments:

1. Section “Search” (page 13): It is laudable that the authors consulted six different libraries, supporting the comprehensiveness of their approach. However, compared to previous reviews, the search strategy seems rather incomprehensive, and some key terms may be missing. Such search terms may include but are not limited to exergaming (MeSH term and all related subterms), X-box, video games, game-based, gamification, gamified, smartphone, television, interactive computer game for the intervention, and mild neurocognitive disorder and related terms for the population. As a result, the sensitivity of the search strategy in identifying all relevant literature is potentially insufficient. This impression seems to be supported by the low number of identified records for such a broad review compared to similar reviews in the field and has also been acknowledged in part by the authors in the Discussion (lines 667-684 that explain this observation with a comparison of eligibility criteria, rather than the sensitivity of the search strategy).

   Good practice would be to pilot test the search strategy to ensure that it identifies known key publications that are consistent with the research question and eligibility criteria (see the Cochrane Handbook for Systematic Reviews of Interventions; https://training.cochrane.org/handbook). I recommend clarifying whether this recommendation has been followed and what the outcome of this process was (e.g., was the search strategy deemed sensitive enough and/or was the search strategy refined?). I strongly recommend to clarify how the search strategy developed, to consult a librarian to update the search strategy following Cochrane Handbook, and transparently report on the results from the search of each library.

2. Classification of Exergame Technology: The manuscript would benefit from clarifying the classification and interpretation of exergame-based interventions. According to the aim and eligibility criteria, this review concerned cognitive and psychosocial interventions. However, the results report on exergame as a “physical training”, which appears to be outside the scope of this review. Nevertheless, since exergame-based training typically targets physical and/or motor-cognitive training, I agree with the authors that this intervention modality should be within the scope

of this review. Therefore, I recommend updating the search strategy to specifically include exergames, clarifying how exergame-based interventions were categorized, and reconsidering (or explaining why you did not) the use a separate category for exergame technologies. Furthermore, the reporting of results should be aligned with the defined objectives (specifically include physical and/or motor-cognitive training modalities). I recommend consulting the following publications to derive methodological choices (particularly regarding the search strategy and categorization) from: https://doi.org/10.3389/fnagi.2022.859715, https://preprints.jmir.org/preprint/65746, https://doi.org//10.1002/14651858.CD013853.pub2, https://doi.org/10.1016/j.arr.2024.102385, or https://doi.org/10.1038/s41746-024-01142-4).

3. Criteria for Technology Categories: The authors note that many studies implemented multiple different technology modalities. To enhance the reproducibility of this review, the manuscript would benefit from providing the criteria that were employed to define the eight categories of technologies analyzed and the specific criteria on how categorization was handled in cases where multiple categories matched.
4. Search results and selection: The manuscript would benefit from providing your results on the inter-rater agreement for all steps of this work that were performed by two independent reviewers (including screening and data extraction), for example by calculating Cohen's kappa, to make more transparent how good the agreement was in these procedures. Please also provide a rationale for the large number of reviewers in relation to the small number of records screened and discuss the limitations of your approach (potentially reduced inter-rater reliability due to the number of reviewers and data extractors involved) in the "Limitations" section.
5. Section "Synthesis" (Page 16 ff) as well as lines 529-538 and 597-604 describe methods of this review and should therefore be moved to the Methods section. In this regard, please also clarify how pilot, feasibility, and qualitative studies were handled in the exploration of effectiveness, given that such studies are not designed to be powered for robust evaluations of an intervention's effects. Furthermore, I recommend also reporting on the observed effect sizes and confidence intervals to provide insight into the clinical meaningfulness of these effects, as solely relying on p-values might not provide a complete picture of the evidence of efficacy given the inclusion of pilot, feasibility, and qualitative studies.
6. Focus on Technology Component: The manuscript puts a heavy focus on the technology part of an intervention. In my view, conceptual decisions and algorithmic decision trees in the training concept together with purpose-developed software build the heart of every digital health technology-enhanced training, whereas it is well-justified to rely on well-established and commercially available hardware as a means to an end to deliver the training to promote accessibility of the resulting intervention. This viewpoint aligns with the "intervention first" rather than a "product first" approach suggested by Torre and Temprado (https://doi.org/10.3389/fnagi.2022.859715). Therefore, I strongly recommend providing more nuance regarding the design and delivery of the interventions that were supported by the technology. Torre and Temprado's paper provides good examples of how this could be achieved and I recommend using this or similar previous contributions as a groundwork to build on.
7. Limitations: The first limitation regarding the exclusion of all "non-novel" technologies used for interventions requires further explanations. From this limitation, it remains unclear which aspects (of software and/or hardware) of the technologies required novelty to be included in this review. As an example, the authors reported that the included studies used different types of VR headsets, including the Oculus Go or HTC Vive, both of which refer to off-the-shelf headsets. The same also goes for screens or other devices (e.g., exergame hardware like Wii Balance Board or Wii Remote) to deliver the intervention. Therefore, the manuscript requires clarification on which exact criteria led to the inclusion of the mentioned examples and particularly the exclusion of this large amount (88 papers) of potentially relevant studies.

   In this regard, I strongly recommend rethinking this methodological choice. While the authors justified this methodological choice regarding exploring trends in research, none of the research questions were formulated accordingly and imply comprehensiveness by covering all existing relevant literature. This mismatch might be misleading to the readers and potentially leads to biased

findings, particularly regarding the efficacy analyses. To better align the methodology with the existing research objectives, I recommend including all technology-enhanced interventions relevant to provide a comprehensive answer to the research questions, and addressing trends as part of a sub-analysis to provide further depth to the findings.

3 Minor Comments:

8. I recommend providing a justification for conducting a scoping instead of a systematic review.
9. Context of VR Applications: The manuscript would benefit from clarifying whether the context of this study was limited to "immersive" VR applications or whether other forms of VR as well as AR and XR were also included. This clarification is especially important in the context of exergame-based training, where most research has focused on non-immersive VR applications like video games displayed on a screen and now more and more AR applications, since VR poses some risks (e.g., for falls) when exercises are performed in a standing position.
10. Implications for Research: The discussion would benefit from providing a dedicated section "Implications for Research" and providing clearer recommendations on how future research can benefit from and build on the findings obtained.
11. Handling of Insufficiently Reported Data: Please report on how the limitation *"In relation to the quality of the studies, many studies did not report the information we needed to answer our research questions (e.g., implementation environment and adherence)"* was handled. Were authors contacted (and how many times) to obtain the insufficiently reported data required in this work?
12. Several sections in the results are interpretative and therefore belong to the discussion section, including: *"This may suggest that the majority of the available digital technologies for cognitive/psychosocial interventions with PwD and MCI may not be yet ready for remote individual home use."* (Page 21, lines 458 - 460) or "This could suggest that some technologies are being designed for a particular group of patients" (page 21, lines 473 - 474)
13. Page 24, lines 587 - 859: I recommend distinguishing between attendance and duration adherence, as these refer to different constructs.
14. Page 12, line 169: The manuscript would benefit from clarifying which criteria the judgement whether the record was based on a "prestigious/reputable conferences (e.g. IEEE-based) with extended papers".
15. Page 12, line 170: The manuscript would benefit from providing a rationale for the 2012 limit.
