# Supplementary material for: The use of digital technology in non-pharmacological cognitive and psychosocial interventions for people with dementia and mild cognitive impairment: A scoping review
Source: PLoS One. 2026 Apr 30;21(4):e0346008. doi: 10.1371/journal.pone.0346008 (PMC13132441; doi:10.1371/journal.pone.0346008)
Supplement: Attachment — Submitted filename: Peer-Review-Report_PONE-D-25-01664_R1.pdf [file pone.0346008.s007.pdf]

Peer Review Report to Manuscript 'PONE-D-25-01664_R1' - The Use of Digital Technology in Non-Pharmacological Cognitive and Psychosocial Interventions for People with Dementia and Mild Cognitive Impairment: A Scoping Review

1 Overview:

I appreciate the opportunity to review this revised manuscript and would like to thank the authors for their time and effort in addressing my initial evaluation. In my original review, I noted that the manuscript addresses a relevant research gap and presents an impressive volume of analyzed articles with findings that could meaningfully inform future research. Based on this, I had anticipated that the authors would be committed to implementing the recommendations provided to strengthen the completeness, methodological rigor, and overall impact of the review.

However, upon examining the response letter and the revised manuscript, it appears that while several comments were addressed thoughtfully, others appear to have been only partially incorporated or not reflected in the manuscript. Specifically, the authors provided a satisfactory response and made the necessary revisions for comments 4, 7, 9, 12 (as far as can be judged without visible track changes), and 15. Comments 2 and 10 were partially addressed, though there may still be opportunities to further enhance the reporting of results (comment 2) and to deepen the discussion in the Implications section (comment 10), particularly by integrating more nuanced reasoning and contextual links to the existing literature. For the remaining comments - nearly half of the total - the authors provided rebuttals; however, I was unable to identify corresponding revisions in the manuscript or its methods.

Most notably, my primary concern continues to center on the sensitivity of the search strategy and the screening methodology, which are foundational to the validity of a scoping review.

To elaborate, the review identified only 1982 records through the database search, which initially raised my concerns about the sensitivity of the search strategy. To assess whether this skepticism is substantiated by the current state of the evidence, I compared the review's findings to those of recent reviews in related domains. According to Figure 3 of the manuscript, among others, 26 studies using gamified technologies, 26 studies with app-based interventions, 18 studies with desktop PC-based interventions, and 16 studies with VR-based interventions were included. In comparison, recent literature suggests that more relevant studies exist in the MCI or dementia population, given the observation that for each of these categories there are reviews available that identified more articles despite a narrower focus on specific populations and outcomes. For example:

<u>Gamified technologies:</u>

- Gao and Liu (2025) [1] identified 28 RCTs in the subcategory of "serious game-based interventions" targeting cognitive performance, although they restricted their analysis on MCI and serious games (which currently only account to about half of interventions in the exergaming research field).

<u>App-based interventions:</u>

- Silva et al (2024) [2] identified 34 studies in the subcategory of "app-based cognitive interventions" in their scoping review.
- Bateman et al. (2017) [3] identified 24 studies in the subcategory of "mobile app-based interventions" for individuals with MCI or dementia, although they restricted their analysis to community-dwelling populations and cognitive performance while this review is over 8 years old.

<u>PC-based interventions:</u>

- Chan et al. (2024) [4] identified 35 RCTs in the subcategory of "computerized cognitive training" in MCI and dementia, although they restricted their analyses to memory outcomes.

- Zuschnegg et al. (2023) [5] identified 24 RCTs in the subcategory of "computer-based interventions" for individuals with MCI or dementia, although they restricted their analysis to community-dwelling populations and cognitive performance

VR-based interventions:

- Chan et al. (2024) [6] identified 20 RCTs in the subcategory of "virtual-reality-based [serious and recreational] exergaming" , although they restricted their analysis to cognitive performance.

While some overlap in primary studies across these reviews is to be expected, this aligns with the double- or multi-categorization applied in the current scoping review. The observation that these reviews identified a similar or greater number of articles - despite narrower research questions (e.g., specific populations or outcomes) - supports my concern that this review may have missed relevant studies due to limitations in the search strategy.

Given the authors' decision not to revise the search strategy as recommended - despite its central importance to the validity of the review's findings - these unresolved concerns may limit the review's comprehensiveness and reliability. While I appreciate the ambition of addressing such a broad and timely research question, reviews of this nature carry considerable weight in academic discourse and must therefore adhere to the highest standards of methodological rigor. For this reason, I believe the manuscript would benefit from further methodological refinement before it can be considered for publication. Should the authors be willing to refine the search strategy and re-screen the literature accordingly, I would be open to re-evaluating a substantially revised version.

2 References:

1. Gao Y, Liu N. Effects of digital technology-based serious games interventions for older adults with mild cognitive impairment: a meta-analysis of randomised controlled trials. Age and Ageing. 2025;54(4):afaf080. doi: https://doi.org/10.1093/ageing/afaf080.
2. Silva AF, Silva RM, et al. Cognitive Training with Older Adults Using Smartphone and Web-Based Applications: A Scoping Review. The Journal of Prevention of Alzheimer's Disease. 2024;11(3):693-700. doi: https://doi.org/https://doi.org/10.14283/jpad.2024.17.
3. Bateman DR, Srinivas B, et al. Categorizing Health Outcomes and Efficacy of mHealth Apps for Persons With Cognitive Impairment: A Systematic Review. Journal of Medical Internet Research. 2017;19(8):e301. doi: https://doi.org/10.2196/jmir.7814.
4. Chan ATC, Ip RTF, et al. Computerized cognitive training for memory functions in mild cognitive impairment or dementia: a systematic review and meta-analysis. npj Digital Medicine. 2024;7(1):1. doi: https://doi.org/10.1038/s41746-023-00987-5.
5. Zuschnegg J, Schoberer D, et al. Effectiveness of computer-based interventions for community-dwelling people with cognitive decline: a systematic review with meta-analyses. BMC Geriatrics. 2023;23(1):229. doi: https://doi.org/10.1186/s12877-023-03941-y.
6. Chan JYC, Liu J, et al. Exergaming and cognitive functions in people with mild cognitive impairment and dementia: a meta-analysis. npj Digital Medicine. 2024;7(1):154. doi: https://doi.org/10.1038/s41746-024-01142-4.
