# Supplementary material for: The use of digital technology in non-pharmacological cognitive and psychosocial interventions for people with dementia and mild cognitive impairment: A scoping review
Source: PLoS One. 2026 Apr 30;21(4):e0346008. doi: 10.1371/journal.pone.0346008 (PMC13132441; doi:10.1371/journal.pone.0346008)
Supplement: Attachment — Submitted filename: ResponseToReviewers.pdf [file pone.0346008.s008.pdf]

Response to the reviewers

Note, all lines references refer to the tracked changes document.

Reviewer 1

Reviewer 1

(of 15 previous responses) - deems "satisfactory" 4,7,9,12,15; 2 and 10 partially addressed

1.Section “Search” (page 13):
1.It is laudable that the authors consulted six different libraries, supporting the comprehensiveness of their approach. However, compared to previous reviews, the search strategy seems rather incomprehensive, and some key terms may be missing.
2.Such search terms may include but are not limited to exergaming (MeSH term and all related subterms), X-box, video games, game-based, gamification, gamified, smartphone, television, interactive computer game for the intervention, and mild neurocognitive disorder and related terms for the population. As a result, the sensitivity of the search strategy in identifying all relevant literature is potentially insufficient.
3.This impression seems to be supported by the low number of identified records for such a broad review compared to similar reviews in the field and has also been acknowledged in part by the authors in the Discussion (lines 667-684 that explain this observation with a comparison of eligibility criteria, rather than the sensitivity of the search strategy).
4. Good practice would be to pilot test the search strategy to ensure that it identifies known key publications that are consistent with the research question and eligibility criteria (see the Cochrane Handbook for Systematic Reviews of Interventions; https://training.cochrane.org/handbook). I recommend clarifying whether this recommendation has been followed and what the outcome of this process was

Response

We thank the reviewer for the detailed feedback once again. We focus on the comments that are considered only partially addressed or not addressed.

1. In lines 172-209 we now provide a description of our revised search strategy. This includes an n-gram (1-2 word) iterative search (using the TERA word frequency tool) for updating the original "seed" string.

2. We include MeSH terms as control terms in our seed string and updated the search string according to our evaluation of the ouput of the n-gram search. Subsequent updates concerned the "free text" high level terms that pertained to technologies. Since our approach focused on novel technologies and so we wanted to start "high level", using the n-gram approach helped us identify terms for specific technologies. Certain terms such as exergaming only came up in the word frequency search in low frequency and adding to the string did not affect the number of documents found in Scopus (the database we used for the word/term frequency search). This was true also of serious games.

3. The identified records have grown from 1646 pre-screened items after removing duplicates (see Prisma diagram Fig 1 of previous submission) to 2654 items after duplicates were removed (see Prisma diagram / Fig 1 of new submission). This compares to the related Sohn et al. (2023) article's identifying 1109 pre-screened items. Our search strategy therefore identified over twice as many pre-screened items as Sohn et al. where we searched from 2012-2025 and Sohn et al. search ed from 2002-2023. However, due to our fairly strict exclusion criteria included studies only rose from 88 to 117, where Sohn et al. (2023) extracted 135 articles. Additional papers in areas such as robotics were found but almost none met our inclusion criteria.

4. In accordance with JBI recommendations for a 3 step search approach, we piloted search strings

(described as step 1 in lines 172-174 and 180-193) and identified controlled and free text terms for our initial search string. We then applied the word/n-gram frequency search to refine the final search string (step 2). Some papers of which we are aware, for example, in robotics, were found but later did not meet our inclusion criteria.
We additionally comment in the text (lines 208-209) "We ran additional searches using known terms like exergame, exergaming and serious games, but this did not affect the terms found in the n-gram frequency search."

2.Classification of Exergame Technology:
1. The manuscript would benefit from clarifying the classification and interpretation of exergame-based interventions. According to the aim and eligibility criteria, this review concerned cognitive and psychosocial interventions. However, the results report on exergame as a "physical training", which appears to be outside the scope of this review. Nevertheless, since exergame-based training typically targets physical and/or motor-cognitive training, I agree with the authors that this intervention modality should be within the scope of this review. Therefore, I recommend updating the search strategy to specifically include exergames, clarifying how exergame-based interventions were categorized, and reconsidering (or explaining why you did not) the use a separate category for exergame technologies.
2. Furthermore, the reporting of results should be aligned with the defined objectives (specifically include physical and/or motor-cognitive training modalities). I recommend consulting the following publications to derive methodological choices (particularly regarding the search strategy and categorization) from:
https://doi.org/10.3389/fnagi.2022.859715,
https://preprints.jmir.org/preprint/65746,
https://doi.org//
10.1002/14651858.CD013853.pub2,
https://doi.org/10.1016/j.arr.2024.102385, or
https://doi.org/10.1038/s41746-024-01142-4).

(2nd review: "Comment 2 ... [was] partially addressed, though there may still be opportunities to further enhance the reporting of results")

3.Criteria for Technology Categories:
1. The authors note that many studies implemented multiple different technology modalities. To enhance the reproducibility of this review, the manuscript would benefit from providing the criteria that were employed to define the eight categories of technologies analyzed …
2. and the specific criteria on how categorization

1. The criteria for defining technologies were agreed upon by the research team, similarly to how we defined the categories for "study setting". Four of the authors are experts in digital technologies and formulated these categories based on their expertise. The definitions for each technology category can be found in Table 2.
In the case of multiple categories, technologies were attributed to both categories e.g: "Gamified

was handled in cases where multiple categories matched.

tasks were often combined with virtual reality, such as the virtual supermarket task in Zhu et al. (2022), or cognitive exergaming paired with VR tasks in Na et al. (2025). In the large 2-stage SMART RCT by Yu et al. (2025), a mobile CT platform was paired with dual-task Tai Chi delivered through either VR or offline instruction, showcasing a hybrid combination of APP, VR, and physical training modalities" (571-577). In these instances, there was a one-to-many mapping between study & technologies.

Additionally, in cases where there was potential ambiguity in interpreting the type of technology and to what category of ours it conformed to, we added (575-577): "In cases of potential ambiguity regarding the type of technology, we assigned terms consistent with those used by the article authors as opposed to reinterpreting them."

Na S, Lee SK, Lee TK, Hong D, Lee ES. The effectiveness of VR-based cognitive training program for mild cognitive impairment: a pilot study. Dementia and Neurocognitive Disorders. 2025 May 21;24(3):174.

Yu C, Bian D, Zhang J, Han X, Shi C, Li G. Effect of Dual-Task Training on Cognitive Function in Community-Dwelling Older Adults With Mild Cognitive Impairment: Sequential Multiple Assignment Randomized Trial. Journal of Medical Internet Research. 2025 Nov 25;27:e79274.

Zhu K, Zhang Q, He B, Huang M, Lin R, Li H. Immersive virtual reality–based cognitive intervention for the improvement of cognitive function, depression, and perceived stress in older adults with mild cognitive impairment and mild dementia: pilot pre-post study. JMIR Serious Games. 2022 Feb 21;10(1):e32117.

5.
1. Section “Synthesis” (Page 16 ff) as well as lines 529-538 and 597-604 describe methods of this review and should therefore be moved to the Methods section.
2. In this regard, please also clarify how pilot, feasibility, and qualitative studies were handled in the exploration of effectiveness, given that such studies are not designed to be powered for robust evaluations of an intervention's effects.

Thank you for the comment. To address this comment and a related comment below of R#2, we have decided to eliminate the boxplots (figures 7-9 from original manuscript, and the relevant text in the MS) synthesizing quantitatively effectiveness per technology/population by plotting the percentage change. Initially we intended to visually explore effectiveness and to identify some patterns. However, we agree with R#2 that given the great heterogeneity across

3.Furthermore, I recommend also reporting on the observed effect sizes and confidence intervals to provide insight into the clinical meaningfulness of these effects, as solely relying on p-values might not provide a complete picture of the evidence of efficacy given the inclusion of pilot, feasibility, and qualitative studies.

studies this may mislead readers, since this is a Scoping Review, and we are not conducting a quantitative analyses in the form of menta-analysis (R#1), we believe that the best way to explore effectiveness is to qualitatively summarise the results presented in the studies included as we did in Table 1, and summarize and discuss our observations based on these qualitative synthesize and the existing literature.

6.Focus on Technology Component:
1. The manuscript puts a heavy focus on the technology part of an intervention. In my view, conceptual decisions and algorithmic decision trees in the training concept together with purpose-developed software build the heart of every digital health technology-enhanced training, whereas it is well-justified to rely on well-established and commercially available hardware as a means to an end to deliver the training to promote accessibility of the resulting intervention. This viewpoint aligns with the "intervention first" rather than a "product first" approach suggested by Torre and Temprado (https://doi.org/10.3389/fnagi.2022.859715).
2.Therefore, I strongly recommend providing more nuance regarding the design and delivery of the interventions that were supported by the technology. Torre and Temprado's paper provides good examples of how this could be achieved and I recommend using this or similar previous contributions as a groundwork to build on.

We are not sure how to address these concerns other than to refer to our previous comments:

"1.We thank the reviewer for this perspective and the article provided for comparison, In Torre and Temprado's paper, we note that they refer to: stimuli, settings, targets, markers, outcomes, moderators, and mechanisms as a structured framework built around seven interacting constructs to put training first. This article, however, is focused on exergaming, whereas our approach is on novel technologies, per se; notwithstanding, we have described/discussed settings, targets, and outcomes, (and moderators to an extent, since we discuss dose, adherence, supervise/unsupervise (which are potential moderators). **See table 1.**
2.The only aspects we do not discuss (from Torre and Temprado's paper) are stimuli, markers and mechanisms, but we believe that, particularly, markers and mechanisms are not the focus of our research questions, and in terms of stimuli, we believe there is too much diversity to account for (not "within the constraints of time and resources", JBI). We do discuss many details of the interventions (settings, targets, several moderators) and tabulate these."

8.I recommend providing a justification for conducting a scoping instead of a systematic review.

We now state explicitly in the text (145-147) "The focus on novelty requires as a first step a scoping review before questions of effectiveness befitting of a systematic review can be investigated."

10.Implications for Research: The discussion would benefit from providing a dedicated section "Implications for Research" and providing clearer recommendations on how future research can benefit from and build on the findings obtained.

(2nd review: "10 [was] partially addressed, though there may still be opportunities to ... to

We have added some text from 1157-1165 (essentially doubled in length this sub-section). In the dedicated section on implications for research, we provide four different recommendations for future research based on our observations and the gaps identified.

deepen the discussion in the Implications section (comment 10), particularly by integrating more nuanced reasoning and contextual links to the existing literature"	
11.Handling of Insufficiently Reported Data: Please report on how the limitation “In relation to the quality of the studies, many studies did not report the information we needed to answer our research questions (e.g., implementation environment and adherence)” was handled. Were authors contacted (and how many times) to obtain the insufficiently reported data required in this work?	We have attempted contact (through email) to a number of authors of papers to provide more information but so far have not received any feedback.
13.Page 24, lines 587 - 859: I recommend distinguishing between attendance and duration adherence, as these refer to different constructs.	We are not sure how to address this point beyond the response we made in our previous resubmission: "*Most studies did not distinguish between attendance versus durations*." The terms were mostly entangled or conflated.
14.Page 12, line 169: The manuscript would benefit from clarifying which criteria the judgement whether the record was based on a “prestigious/reputable conferences (e.g. IEEE-based) with extended papers”.	Note we have deleted line 231 "or prestigious/reputable conferences (e.g. IEEE-based) " as reviewers agreed that this was not used as an inclusion/exclusion criterion in practice.
**Reviewer 2**	We thank the reviewer for the comments and address one by one below.
(1) Insufficient methodological transparency? in screening/extraction processes (e.g., reviewer roles, conflict resolution, application of subjective "novelty" criteria, handling of overlapping technologies like GT/APP);	Reviewer roles: In lines 271-274 we have written "*The references identified from the title and abstract screening search phase and after removing duplicates using Covidence, were n=2654 (see Fig 1). Six of the authors (QL, SN, PG, AV, MD, IK) were involved in this review, and a pair of two reviewers reviewed each full-text reference. Another author (RL) resolved all the conflicts.*" In 224-226 we have written: "*Inter-rater agreement per stage, as checked in Covidence (Covidence 2022), achieved 84% for Title and Abstract screening (8 reviewer pairs) and 74% for Full text screening (6 reviewer pairs). Furthermore, 6 reviewers were used for extraction and 3 reviewers for final consistency checks.*"<br><br>We define novelty in lines 254-268: "*We adopted the below 3 criteria for evaluating whether the technology was to be excluded due to non-novelty. These criteria were decided based on a workshop carried out at the start of the project (April 26th, 2022):*<br><br>1. *Not novel digitalization or “novel application”: e.g. beyond basic PC "interface" for*

Psychology task:

- *do not exclude "old" digitalization approach that has new application;*
- *do not exclude "old" digitalization approach that is at increased maturation stage, e.g. new FDA approved example;*

2. *Not a temporary application: e.g. distance-based method, possibly due to the COVID-19 pandemic, and isn't intended as a longer term solution;*
3. *Not just a computer screen interface: digital technology can be software or hardware but not simply a computer screen unless novel software (or application) is used, i.e. point 1.*

We define novel application as: technology that produces original data through a pilot study, intervention, validation study, or feasibility study, which aims to improve psychosocial function, cognition, or well-being in patients."

For handling overlapping technology, we have now added (575-577): "In cases of potential ambiguity regarding the type of technology, we assigned terms consistent with those used by the article authors as opposed to reinterpreting them."

"(2) ?Problematic presentation and interpretation of results, particularly the oversimplified quantitative synthesis (Figs 7-9) using a "percentage change" metric that masks critical heterogeneity in outcomes, measures, and study designs, potentially leading to misleading conclusions about technology efficacy;

Thank you for the comment. To address this comment and a related comment above of R#1, we have decided to eliminate the boxplots (figures 7-9 from original manuscript, and the relevant text in the MS) synthesizing quantitatively effectiveness per technology/population by plotting the percentage change. Initially we intended to visually explore effectiveness and to identify some patterns. However, we agree that given the great heterogeneity across studies this may mislead readers, since this is a Scoping Review, and we are not conducting a quantitative analyses in the form of menta-analysis (R#1), we believe that the best way to explore effectiveness is to qualitatively summarise the results presented in the studies included as we did in Table 1, and summarize and discuss our observations based on these qualitative synthesize and the existing literature.

We have now eliminated figs (box plots) 7-9 as we feel this may mislead readers into perceiving

the paper to represent a systematic review with quantitative synthesis when in reality we are presenting a scoping review with qualitative synthesis.

(3) ?Inadequate discussion? of the implications of extreme methodological heterogeneity across studies, limitations of the technology classification framework, and potential biases introduced by the novelty-focused inclusion criteria;

We would like to clarify (again in relation to the removed box plots of figures 7-9) that we are not making a quantitative comparison of the type that could be analyzed for systematic review with meta analysis ... we are doing a scoping review so provide indicators only regarding outcomes.

We have revised the discussion in order to address comments from R#1 & R#2 so we hope that reviewers now find it more informative. Indeed there is great heterogeneity across studies, and we have discussed this extensively in the Discussion sections (and in the Limitations and Conclusion subsections). The great heterogeneity observed in our review is also in line with the observations and conclusions of other similar previous reviews (e.g., Kletzel et al., 2021; Zuo et al., 2024), so we have emphasized in our conclusions the need to produce evidence-based guidelines for their implementation of technology-based interventions in healthcare setting. With regard to the bias introduced by the novelty criterion, please see responses to R#1 for the justification of this criterion. We have discussed extensively the limitations of this criterion in the Discussion section.

Kletzel SL, Sood P, Negm A, Heyn PC, Krishnan S, Machtinger J, Hu X, Devos H. Effectiveness of brain gaming in older adults with cognitive impairments: a systematic review and meta-analysis. Journal of the American Medical Directors Association. 2021 Nov 1;22(11):2281-8.

Zuo X, Tang Y, Chen Y, Zhou Z. Effects of Electronic Serious Games on Older Adults With Alzheimer’s Disease and Mild Cognitive Impairment: Systematic Review With Meta-Analysis of Randomized Controlled Trials. JMIR Serious Games. 2024 Jul 31;12(1):e55785.

(4) ?Critical formatting issues? in core tables (e.g., Table 1 data misalignment). Addressing these is essential for scientific rigor.

We assume the article will be formatted, if it is accepted, according to double columns, etc., but if this is not the case we are happy to correct.

Reviewer 3

No issues reported

We thank the reviewer for the time and initial feedback.
