# Supplementary material for: The use of digital technology in non-pharmacological cognitive and psychosocial interventions for people with dementia and mild cognitive impairment: A scoping review
Source: PLoS One. 2026 Apr 30;21(4):e0346008. doi: 10.1371/journal.pone.0346008 (PMC13132441; doi:10.1371/journal.pone.0346008)
Supplement: Attachment — Submitted filename: Dr.Suad-Ghaben-PONE-25-01664.docx [file pone.0346008.s006.docx]

**Dr. Suad Ghaben**

**PONE-25-01664**

**The Use of Digital Technology in Non-Pharmacological Cognitive and Psychosocial Interventions for People with Dementia and Mild Cognitive Impairment: A Scoping Review.**

Dear Authors

Please find my comments on your manuscript below. Your manuscript is a scoping review on the Use of Digital Technology in Non-Pharmacological Cognitive and Psychosocial Interventions for People with Dementia and Mild Cognitive Impairment. Your results highlighted eight main categories of digital technologies that has been utilized within the study timeframe mainly gamified technologies, mobile app, web-based approaches, and virtual reality. Positive cognitive outcome was the most detected outcome compared to functional and well-being outcomes.

**I will start by commenting on the manuscript parts in order highlighting major and minor revisions.**

**Title:**

**Short, concise, and reflective. No comments**

**Abstract:**

**Major:**

- Add to the methods\design section how screening was performed.
- Add to the methods section “We followed the Joanna Briggs Institute guidelines and the framework”.
- Add to the results section the number of studies you analyzed.

**Minor:**

- Use objective instead of background to align with the journal style.
- No need to mention the results of the geographic data in the results section.
- Replace “our study” with “this study” in the results and discussion sections

**Introduction:**

**Major:**

- Suggest to add subheading “Literature review”
- 98-99: cite
- 100-102: cite
- Lines 84-85: suggest to search literature on bibliometric studies on the topic and cite accordingly.
- Lines 117-128: be specific in identifying the research gap.

**Minor:**

- Line 55: PWD: spell out in full on first mention
- Line 103: deployment/implementation: this style of writing is not accepted in academia. Rather you can use one term or describe each term separately.

**Methods:**

**Major:**

- Rearrange the methods section and add the mentioned subheadings in order: search strategy, eligibility criteria, study selection, data extraction and synthesis.
- The methods section lacks the main description of the followed methodology, which is mostly described under the results section.

**Minor:**

- Lines 224-225: this is consistent with the findings … move this interpretation to the discussion section. Result section is to present your results only.

**Results:**

**Major:**

- rearrange the results section
- add the following subheadings: Study methods, study characteristics, characteristics of digital interventions, effectiveness outcomes
- lines 133-235 : data charting process ”Four team members … consistency of the items extracted. Move this part to the methods section under the data extraction and synthesis subheading .. and under the “Study characteristics” subheading under the “Results” section describe the end user population, geographic location and funding sources .. etc.
- lines 288-289, 417-419, 483-484, 518-519: delete the RQs and describe the answers for each question in separate paragraphs.
- 275-286: can you justify the deployment of the PICO framework? the PICO framework was not included as an eligibility criterion.
- Any description related to the applied farmwork should be moved to the Methods section.

**Minor:**

- keep consistency of the methods and results sections

**Discussion:**

**Major:**

- lines 640-652: no need to describe the research gap here. Rather you need to go straight summarizing your main results.
- Line 898: Other gaps in knowledge… the discussion section is to interpret your results and compare it with existing literature... rather than discussing the knowledge gap. It can be accepted to discuss the knowledge gap only if your goal is to find the research gap in digital technologies ….. etc.

**Minor:**

- Lines 944-946: what are the criteria of considering the digital interventions as novel? This was not described in the eligibility criteria and exclusion criteria which could jeopardize the mentioned methodology and results.
- Lines 955-960: evaluating the quality of studies is not a prerequisite in scoping review. Thus; delete this paragraph.

**Conclusion:**

**Major:**

- Lines: 985-994: no need to describe the rational of conducting this review. Which was described in full in the introduction. Delete this paragraph.
- Lines: 995-1017: the conclusion is to restate the RQs and summarize the key findings results rather than redescribing the key findings or contrasting them with existing literature. Rewrite this section focus on summarize the key findings of this review, delete other information related to other literature.

**General comments:**

- The manuscript should be rewritten in a concise scientific language.
- The manuscript is disorganized; it should be rearranged in headings and subheadings that enhance its flow and readability and aligns with the Journal guidelines.
- Citation style is not aligned with the journal guidelines.
- the manuscript is wordy with a lot of redundancy; suggest to write in terse concise language.
- The manuscript should be thoroughly revised; some information missed in its section, however, described in wrong section.
